# Supplementary material for: Obtaining a Monoclonal Antibody against a Novel Prometryn-Like Hapten and Characterization of Its Selectivity for Triazine Herbicides
Source: Biosensors (Basel). 2022 Dec 25;13(1):22. doi: 10.3390/bios13010022 (PMC9855386; doi:10.3390/bios13010022)
Supplement: Supplementary file 1 [file biosensors-13-00022-s001.zip › biosensors-2016910-supplementary.pdf]

Table S1. Serum potency and inhibition rate in mice.

Immunization cycle	Dilution of immunogen	$1×10^3$			$2×10^3$			$4×10^3$			$8×10^3$		
	(Serum dilution)	C	I	IR	C	I	IR	C	I	IR	C	I	IR
**Third immunization**	$4×10^3$	3.4987	0.6147	82%	3.2217	0.4268	87%	1.957	0.2932	85%	1.0275	0.273	73%
	$8×10^3$	2.0517	0.3646	82%	1.4623	0.2116	86%	1.0487	0.2047	80%	0.5481	0.1797	67%
	$16×10^3$	1.3106	0.2041	84%	0.9662	0.1787	82%	0.7009	0.1313	81%	0.3971	0.1131	72%
	$32×10^3$	0.7766	0.1608	79%	0.4838	0.1299	73%	0.3656	0.1268	65%	0.2269	0.1149	49%
**Immunization cycle**	**Dilution of immunogen**	$1×10^3$			$2×10^3$			$4×10^3$			$8×10^3$		
	**(Serum dilution)**	**C**	**I**	**IR**	**C**	**I**	**IR**	**C**	**I**	**IR**	**C**	**I**	**IR**
**Fourth immunization**	$16×10^3$	3.3663	0.4319	87%	3.3583	0.3121	91%	2.7764	0.2348	92%	1.9664	0.1995	90%
	$32×10^3$	3.1291	0.2163	93%	2.6928	0.1715	94%	1.7124	0.1501	91%	1.2114	0.1324	89%
	$64×10^3$	1.6764	0.1533	91%	1.4977	0.139	91%	1.0592	0.1256	88%	0.6699	0.1231	82%
	$128×10^3$	1.1354	0.1301	89%	0.9073	0.1271	86%	0.5824	0.1042	82%	0.3814	0.1043	73%

Note: "C" represents control wells, "I" represents inhibition wells, and "IR" represents inhibition rate. The inhibitory concentration of ATR was 1000 ng/mL.

Table S2. Serum titer and inhibition rate of mice for the fusion experiment.

**Dilution of immunogen**	**1×10^3**			**2×10^3**			**4×10^3**		
**Dilution of Serum**	**C**	**I**	**IR**	**C**	**I**	**IR**	**C**	**I**	**IR**
**2×10^3**	3.6755	0.5255	85.70%	3.7644	0.5594	85.14%	3.5861	0.474	86.78%
**4×10^3**	2.5767	0.3082	88.04%	2.4852	0.3242	86.95%	2.5613	0.3231	87.39%
**8×10^3**	1.0701	0.1984	81.46%	1.1349	0.2317	79.58%	1.0939	0.1998	81.74%
**1.6×10^4**	0.5978	0.2041	65.86%	0.5028	0.1911	61.99%	0.5897	0.2128	63.91%
**3.2×10^4**	0.3286	0.176	46.44%	0.3683	0.1781	51.64%	0.3518	0.184	47.70%
**6.4×10^4**	0.4051	0.2612	35.52%	0.3473	0.3122	10.11%	0.3912	0.2938	24.90%
**1.28×10^5**	0.2482	0.2672	-7.66%	0.2661	0.2283	14.21%	0.2624	0.2424	7.62%
**2.56×10^5**	0.5585	0.1388	75.15%	0.5693	0.1429	74.90%	0.5725	0.1235	78.43%
**Dilution of immunogen**	**8×10^3**			**1.6×10^4**			**3.2×10^4**		
**(Serum dilution times)**	**C**	**I**	**IR**	**C**	**I**	**IR**	**C**	**I**	**IR**
**2×10^3**	3.7084	0.4723	87.26%	3.5442	0.5299	85.05%	3.6483	0.5562	84.75%
**4×10^3**	2.4955	0.3956	84.15%	2.3486	0.4081	82.62%	2.7125	0.8551	68.48%
**8×10^3**	1.2335	0.2933	76.22%	1.3561	0.2508	81.51%	1.4674	0.2825	80.75%
**1.6×10^4**	0.5945	0.2485	58.20%	0.6571	0.2718	58.64%	0.6988	0.1786	74.44%
**3.2×10^4**	0.3317	0.2797	15.68%	0.4686	0.3348	28.55%	0.3682	0.1776	51.77%
**6.4×10^4**	0.3813	0.3515	7.82%	0.4456	0.3958	11.18%	0.423	0.3017	28.68%
**1.28×10^5**	0.3321	0.3083	7.17%	0.2771	0.4234	-52.80%	0.2685	0.2207	17.80%
**2.56×10^5**	0.6379	0.2572	59.68%	0.7063	0.2009	71.56%	0.5793	0.161	72.21%

Note: “C” represents control wells, “I” represents inhibition wells, and “IR” represents inhibition rate. The concentration of the coating substance was 1 mg/mL. The inhibitory concentration of prometryn was 1000 ng/mL.

Table S3. Optimal ic-ELISA combination of coated antigen and antibody.

Dilution of immunogen	1×10^3			2×10^3		
**Dilution of Serum**	**C**	**I**	**IR**	**C**	**I**	**IR**
2×10^3	3.8036	0.0981	97%	3.4246	0.0828	98%
4×10^3	3.3319	0.0819	98%	2.8464	0.0725	97%
8×10^3	2.2213	0.074	97%	1.6744	0.0699	96%
1.6×10^4	1.2133	0.0724	94%	0.9525	0.0788	92%
3.2×10^4	0.8439	0.0709	92%	0.4457	0.0664	85%
**Dilution of immunogen**	**4×10^3**			**8×10^3**		
**(Serum dilution times)**	**C**	**I**	**IR**	**C**	**I**	**IR**
2×10^3	2.1325	0.0622	97%	1.0654	0.0695	93%
4×10^3	1.2547	0.0652	95%	0.6322	0.0646	90%
8×10^3	0.6402	0.0627	90%	0.3649	0.0641	82%
1.6×10^4	0.349	0.065	81%	0.1867	0.0623	67%
3.2×10^4	0.2048	0.06	71%	0.138	0.062	55%

Note: "C" represents control wells, "I" represents inhibition wells, and "IR" represents inhibition rate. The concentration of the coating agent was 1 mg/mL. The inhibitory concentration of prometryn was 1000 ng/mL.
